# Predicting pneumonia mortality using CURB-65, PSI, and patient characteristics in patients presenting to the emergency department of a comprehensive cancer center

**DOI:** 10.1002/cam4.240

**Published:** 2014-05-07

**Authors:** Carmen Gonzalez, Tami Johnson, Kenneth Rolston, Kelly Merriman, Carla Warneke, Scott Evans

**Affiliations:** 1 Department of Emergency Medicine, Division of Internal Medicine, UT MD Anderson Cancer Center1515 Holcombe Blvd., Houston, 77030, Texas; 2 Pharmacy Clinical Programs, UT MD Anderson Cancer CenterHouston, Texas; 3 Department of Infectious Diseases, UT MD Anderson Cancer CenterHouston, Texas; 4 Department of Emergency Medicine, UT MD Anderson Cancer CenterHouston, Texas; 5 Department of Biostatistics, UT MD Anderson Cancer CenterHouston, Texas; 6 Department of Pulmonary Medicine, UT MD Anderson Cancer CenterHouston, Texas

**Keywords:** Cancer, CURB-65, emergency department, pneumonia, PSI

## Abstract

The prognostic accuracy of the CURB-65 criteria and pneumonia severity index (PSI) in immunocompromised cancer patients with pneumonia is unknown. We sought to determine whether CURB-65 and PSI predict 28-day mortality in cancer patients with pneumonia, and identify other factors that predispose cancer patients with pneumonia to a high mortality risk. We assessed sensitivities, specificities, predictive values, and areas under the receiver operating curve area under the curve (AUC) of the CURB-65 and PSI in predicting the 28-day mortality of cancer patients presenting to our institution's emergency department with pneumonia. We used the DeLong and Clarke–Pearson approach to determine whether the addition of other risk factors improved the scales' performances. The overall and pneumonia-related 28-day mortality rates were 20.2% (*n* = 44) and 17.4% (*n* = 38), respectively. In predicting 28-day mortality, the CURB-65 score had sensitivity of 45% and specificity of 81%, and the PSI score had sensitivity of 82% and specificity of 34%. The CURB-65 and PSI discriminated poorly between fatal and nonfatal pneumonia cases (AUCs, 0.664 and 0.658, respectively; 95% confidence interval [CI], 0.57–0.75 for each). The addition of radiation therapy (RT) within 4 weeks and stem cell transplant (SCT) significantly improved the AUCs of the CURB-65 (0.75; 95% CI, 0.67–0.83) and PSI (0.73; 95% CI, 0.65-0.82). Inadequate performances of CURB-65 and PSI demonstrate that a tool for predicting pneumonia-related mortality in cancer patients and other immunocompromised populations is needed. Pneumonia patients who have undergone recent RT or (SCT) are at a high risk of dying from pneumonia and require special consideration when assessing pneumonia-related mortality risk.

## Introduction

Pneumonia is a leading cause of death among cancer patients 1. The characteristics of cancer patients who develop pneumonia differ markedly from those of patients in the community with pneumonia 2. Compared with pneumonia patients without cancer, who tend to develop community-acquired pneumonia (CAP), patients receiving treatment for their cancer or cancer-related complications are more often diagnosed with healthcare-associated pneumonia (HCAP). Cancer patients develop a spectrum of infections that depend on the patients' specific underlying immunologic defects (e.g., immunoglobin, complement, cellular immunity, and granulocyte deficiencies), local barrier defense defects (e.g., mucositis, tumor invasion), and malignancy type (hematologic or solid). In the population with lung cancer, factors such as tumor causing localized airway obstruction and radiation therapy (RT) causing underlying mucosa changes can predispose these patients to the development of pneumonia. In patients who receive stem cell transplant (SCT), other factors can affect the risk of infection, such as graft versus host disease and the type of transplant (allogeneic or autologous) 3.

Cancer patients undergoing treatment who develop a respiratory infection often first present to the emergency department (ED). Therefore, the ED has a very important role in the initial evaluation, assessment, management, and disposition of these patients. One important component of the initial evaluation of patients who present to the ED with pneumonia is pneumonia severity risk stratification. Several scoring systems, including the CURB-65 criteria (**c**onfusion; urea >7 mmol/L; respiratory rate >30 breaths/min; systolic blood pressure <90 mmHg, diastolic **b**lood pressure <60 mmHg; age ≥65 years) and the pneumonia severity index (PSI), are used to help predict pneumonia severity, prognosis, and short-term mortality in patients with CAP 4,5. The CURB-65 6 and the PSI 7 are designed to stratify adults with CAP into their risk of pneumonia-related mortality and management groups. However, these evaluation systems and the associated management guidelines which are widely used have been validated for immunocompetent adults, not immunocompromised patients such as cancer patients 4,8. The PSI has one variable that accounts for neoplastic disease, and two studies reported limited findings regarding the use of the CURB-65 and PSI in patients with HCAP 9,10, but no outcome prediction tools for immunocompromised cancer patients who present with pneumonia have been developed. The purpose of the present study was to determine whether the CURB-65 and PSI predict pneumonia outcomes in cancer patients who presented to the ED of a cancer center. We hypothesized that these tools are not helpful in this setting because most patients have HCAP and/or are immunocompromised. We also sought to identify other factors that predispose cancer patients with pneumonia to a high mortality risk.

## Methods

### Study design and patients

We conducted a retrospective cohort study of consecutive patients who presented with pneumonia to The University of Texas MD Anderson Cancer Center's ED, a 43-bed unit that has 20,000 patient visits each year, in February, October, November, and December 2008. The study sample was drawn from an ongoing quality improvement initiative. Two trained reviewers independently evaluated the patients' medical records to confirm that patients met the criteria for pneumonia. Patients were classified as having HCAP or CAP. Patients' demographic and clinicopathologic data were abstracted from the medical record and entered into a database. MD Anderson's Institutional Review Board approved the study.

Patients aged 18 years or older who had an International Classification of Diseases, Ninth Revision (ICD-9) code of pneumonia were enrolled in the study. Patients were considered to have pneumonia if they had symptoms of an acute lower respiratory tract illness (e.g., coughing) with or without sputum production, or a focal chest sign at auscultation that could be accompanied by systemic symptoms such as fever or chills, and confirmed by the presence of a new infiltrate revealed by chest radiography or computed tomography at the time of ED presentation. Pregnant women, patients with a diagnosis of pneumonia within 7 days of presentation to the ED, and patients with an ICD-9 diagnosis of aspiration pneumonia were excluded from the study. Patients were designated as having HCAP if they had been hospitalized for two or more days within 90 days, resided in a nursing home or extended care facility, received home infusion therapy (including antibiotics), had received chronic dialysis within the last 30 days, received home wound care, and/or had a family member with a multidrug-resistant pathogen 8. Patients who had received nonsurgical cancer therapy in the 4 weeks prior to their pneumonia diagnosis were considered to have HCAP. Patients who were diagnosed with pneumonia that did not fit the criteria for HCAP and did not have hospital-acquired pneumonia were considered to have CAP.

### Variables

Our primary outcome variable was 28-day mortality, which was defined as documented death from any cause during hospitalization or within 28 days of presentation to the ED. Patients who were discharged from the hospital to home or hospice care 28 days after presentation were considered to be alive at the end of follow-up. Deceased patients' medical records and/or death certificates were reviewed to determine causes of death.

Independent variables included patient characteristics such as age and gender and clinical variables, including systemic inflammatory response syndrome (SIRS) status, liver disease, heart failure, cerebrovascular disease, renal disease, altered mental status on arrival to the ED, CURB-65 score, PSI score, type of malignancy, use of steroids, or nonsurgical cancer treatments within 4 weeks prior to ED presentation, and SCT status. Appropriate use of antibiotics per The Infectious Diseases Society of America, The American Thoracic Society and local institutional guidance was evaluated 4,8. patients' CURB-65 and PSI scores were calculated based on data entered into the ED medical record at the time of the patients' initial ED presentation. Patients were stratified into low-, intermediate-, and high-risk groups according to their PSI and CURB-65 scores 6,7. A CURB-65 score ≥2 has been described to indicate an intermediate or high risk of pneumonia-related mortality in other populations, and this rule was used in our study. Microbiology from specimens obtained from blood, sputum, bronchoscopy, and nasal wash as well as their sensitivity were abstracted from the patients' electronic medical record (EMR) and entered into a database.

Patients who had been diagnosed with malignancy, other than nonmelanoma skin cancer in the 12 months before ED presentation and were in active treatment, and patients who had received chemotherapy and/or RT in the 12 months before ED presentation were considered to have active cancer treatment. Patients who received adalimumab, alemtuzumab, fludarabine, infliximab, rituximab, and/or temozolomide in the 2 years before ED presentation were considered to have been exposed to novel agents which included prolonged immunosuppressive chemotherapies and monoclonal antibodies, and patients who had undergone RT for a malignancy in the chest in the 4 weeks before presentation with pneumonia were considered to have received RT. Patients with a neutrophil count of <500 cells/mm^3^ or <1000 cells/mm^3^ that was projected to decrease to <500 cells/mm^3^ were considered to have neutropenia. Patients were considered to have SIRS 11 if they had two or more of the following conditions: hyperthermia (>101°F) or hypothermia (<96°F); tachycardia (heart rate >90 beats per min); tachypnea (respiratory rate >20 breaths per min); or a white blood cell count of >12,000 cells/mm^3^ or <4,000 cells/mm^3^.

### Statistical analyses

We included patients' unique ED visits in the analysis. For patients who presented to the ED twice, we randomly selected one visit to include in the analysis. We used frequencies, percentages, and median values to describe patients' demographic and clinical characteristics. We calculated the sensitivities, specificities, negative predictive values, and positive predictive values of the CURB-65 and PSI for discriminating between fatal and nonfatal cases of pneumonia and the corresponding exact binomial 95% confidence intervals (CIs). The receiver operating characteristics (ROC) area under the curve (AUC) 12 was described for each scoring system. We used multiple logistic regression analysis to determine whether the CURB-65 score, the PSI score, and/or other independent variables (as described above) predicted 28-day mortality. Final regression models were selected using a forward selection procedure. The discrimination capability of the combination of each severity scale with other factors was evaluated in the ROC analysis. The AUCs for the prediction models and their 95% CIs were calculated and compared using the DeLong and Clarke–Pearson approach 13. *P* values were two-tailed and considered significant at *α* < 0.05. All analyses were conducted using the SAS statistical software package for Windows (version 9.2, SAS Institute, Cary, North Carolina).

## Results

### Patient characteristics

We identified 227 pneumonia cases in 218 cancer patients, of whom 209 had one episode of pneumonia and nine had two episodes of pneumonia. The median patient age was 60 years (range, 20–83 years). The primary cancer diagnosis was solid tumors in 121 patients (55.5%). Of those, 44 (36.4%) had a lung malignancy, 18 (14.9%) had a head and neck malignancy, 17 (14%) had a breast malignancy, 16 (13.2%) had a gastrointestinal malignancy, nine (7.4%) had a genitourinary malignancy, and 17 (14%) had other malignancies such as, sarcoma, gynecologic, or melanoma. Hematologic malignancy was the diagnosis in 94 patients (43.1%). Of those, 41 (44%) had leukemia, 15 (16%) had lymphoma, seven (7%) had multiple myeloma, and 31 (33%) had SCT. Three patients (1.4%) did not have active cancer. HCAP was diagnosed in 191 patients (87.6%), and CAP was diagnosed in 27 patients (12.4%). Overall, 44 patients (20.2%) died within 28 days of presenting to the ED; 38 patients (17.4%) died of pneumonia.

### Microbiology

In our data, a total of 52 pathogens were isolated from sputum or bronchoscopy. The patients were categorized by the type of pneumonia in HCAP and CAP groups. The distribution of pathogens varied among the two pneumonia categories (Table1). The majority of those patients had a clinical classification of HCAP; drug-resistant pathogens were identified only in those patients (21%). *Staphylococcus* aureus (*S. aureus*) was the most common pathogen, 46% were methicillin-resistant *S. aureus* (MRSA) and all were identified in the HCAP group. The most common gram-negative bacteria were *Pseudomonas aeruginosa* (24%) and *Haemophilus species* (20%). Of the 218 patients, 191 had at least one set of blood cultures done, only 22 (12%) yielded a positive result. The most predominant organisms were gram-positive bacteria (total of 15) such as *S. aureus and Staphylococus epidermidis* (23% each), followed by *Escherichia coli* (14%) and *P. aeroginosa* (9%). Nasal washes were done in 17% of the patients, yielding a positive culture in 13 specimens. The most common pathogen identified in nasal wash was Respiratory Syncytial Virus (38%) all in patients with hematologic malignancies.

**Table 1:** Distribution of bacterial isolated pathogens between cancer patients with Community-acquired Pneumonia (CAP) and Healthcare-associated Pneumonia (HCAP).^2^

Bacterial pathogens from sputum or bronchoscopy (*n* = 52)	Total (MDR)	CAP	HCAP (MDR)
Gram-positive pathogens	21	2	19
* Staphylococcus aureus*	13	1	12
MSSA	7	1	6
MRSA	6		6
* Streptococcus* species	3		3
* Enterococcus* species	4	1	3
* Nocardia*	1		1
*Mycobacterium* pathogens	6	1	5
* Avium*	1	1	
Other^1^	5		5
Gram-negative pathogens	25	2	23
* Pseudomonas aeruginosa*	6 (2)		6 (2)
* Haemophilus* species	5	2	3
* Klebsiella pneumoniae*	3		3
* Escherichia coli*	4 (2)		4 (2)
* Enterobacter* species	4		4
* Stenotrophomonas maltophilia*	3		3

MDR, multidrug-resistant pathogen; MSSA, methicillin-sensitive *Staphylococcus aureus;* MRSA, methicillin-resistant *Staphylococcus aureus*.

1 Abscessus, 2 Intracellulare, Kansaii, and Tuberculosis.

2 The pathogens in this table may not represent a complete list of potential pathogens as it is based only on cultures.

### Curb-65

CURB-65 scores ranged from 0 to 4 (Table2A). A total of 165 patients (75.7%) had a score of 0 or 1 and were classified as having a low risk of pneumonia-related mortality; of these patients, 24 (14.6%) died within 28 days. Fifty-three patients (24.3%) had a score of 2 or higher and were classified as having an intermediate or high risk of pneumonia-related death; of these patients, 20 (37.7%) died within 28 days. Of the 53 patients in the intermediate- or high-risk group, 47 (88.7%) received pneumonia treatment that was in line with or more cautious than that recommended by the CURB-65 guidelines. Among the six patients who did not receive such treatment, one of the two intermediate-risk patients who were discharged to home or hospice care died, and two of the four high-risk patients who were admitted to the hospital died (Table2A).

**Table 2:** Disposition according to risk level as determined by (A) CURB-65 Score (B) PSI class.

Disposition	Low risk	Intermediate risk	High risk
All patients	*n* = 165	*n* = 42	*n* = 11
Home/hospice care	21 (13)	2 (5)	0
Hospital floor	117 (71)	25 (60)	4 (36)
Telemetry	21 (13)	10 (24)	2 (18)
ICU	6 (4)	5 (12)	5 (45)
Patients who died within 28 days	*n* = 24	*n* = 15	*n* = 5
Home/hospice	0	1 (7)	0
Hospital floor	16 (67)	8 (53)	2 (40)
Telemetry	6 (25)	3 (20)	1 (20)
ICU	2 (8)	3 (20)	2 (40)
	Low risk		High risk
Disposition	Class I	Class II or III	Class IV or V
All patients	*n* = 5	*n* = 63	*n* = 150
Home/hospice care	0	14 (22)	9 (6)
Hospital floor	5	42 (67)	99 (66)
Telemetry	0	4 (6)	29 (19)
ICU	0	3 (5)	13 (9)
Patients who died within 28 days	*n* = 1	*n* = 7	*n* = 36
Home/hospice	0	0	1 (3)
Hospital floor	1	5 (71)	20 (56)
Telemetry	0	1 (14)	9 (25)
ICU	0	1 (14)	6 (17)

All data are number of patients (%). ICU, intensive care unit.

The estimated odds of death within 28 days almost doubled with each point increase in CURB-65 score (odds ratio [OR], 1.85; 95% CI, 1.30–2.62) (Table3). CURB-65 score (<2 vs. ≥2) predicted pneumonia-related mortality with an overall sensitivity and specificity of 45% and 81%, respectively (Table4). ROC analysis revealed that although the CURB-65's diagnostic performance was significantly better than chance (*P *< 0.001), the CURB-65 had only modest utility for discriminating between fatal and nonfatal pneumonia cases (AUC, 0.6635; 95% CI, 0.57–0.75).

**Table 3:** Univariate logistic regression model for predicting 28-day mortality.

Label	Frequencies			Odds ratio estimate		*P*
	Alive	Dead	Total	OR	95% CI	
Patient age category						
≥60 years	92	22	114	0.891	0.460–1.727	0.7332
≤60 years	82	22	104	1.000	0.980–1.028	0.7643
Per year increase	174	44	218	1.004		
Gender						
Female	68	16	84	0.891	0.449–1.768	0.7409
Male	106	28	134	1.000		
Pneumonia severity index						
IV or V	114	36	150	2.368	1.035–5.417	0.0411
I–III	60	8	68	1.000	1.318–3.324	0.0017
Per unit increase	174	44	218	2.093		
CURB-65 score						
CURB-65 ≥ 2	33	20	53	3.561	1.761–7.200	0.0004
CURB-65 ≤ 2	141	24	165	1.000	1.301–2.620	0.0006
Per unit increase	174	44	218	1.846		
Met SIRS criteria	120	35	155	1.750	0.786–3.894	0.1703
Tumor type						
Hematologic	75	19	94	0.973	0.498–1.899	0.9356
Solid	96	25	121	1.000		
Leukemia	35	6	41	0.627	0.246–1.601	0.3292
Absolute neutrophil count, cells/mm³						
<100	12	1	13	0.320	0.040–8.231	0.7406
100 to <499	5	2	7	1.537	0.287–3.655	
500 to <999	10	2	12	0.768	0.162–0.2813	
1000 or greater	146	38	184	1.000	2.540–0.6158	
Steroids	80	20	100	0.979	0.504–1.902	0.9505
Chemotherapy	84	19	103	0.814	0.418–1.586	0.5458
Immunotherapy	11	4	15	1.482	0.449–4.899	0.187
Novel chemotherapy	20	8	28	1.711	0.698–4.194	0.2403
Radiation therapy	9	8	17	4.074	1.472–11.279	0.0069
Stem cell transplant	22	9	31	1.777	0.753–4.191	0.1893
Guideline concordance of antibiotic selection	59	13	72	0.817	0.398–1.679	0.5829
Pneumonia classification						
HCAP	151	40	191	1.523	0.498–4.657	0.4605
CAP	23	4	27	1.000		

OR, odds ratio; CI, confidence interval.

**Table 4:** Sensitivity, specificity, and positive and negative predictive values of the CURB-65 and PSI in predicting the 28-day mortality.

Value % (95% CI)	CURB-65	PSI^1^
Sensitivity	45 (30–61)	82 (67–92)
Specificity	81 (74–87)	34 (27–42)
PPV	38 (25–52)	24 (17–32)
NPV	85 (79–90)	88 (78–95)

CI, confidence interval; PPV, positive predictive value; NPV, negative predictive value.

1 PSI class I, II, or III versus IV or V.

### Psi

The median PSI class was IV (Table2B). The 28-day mortality rates of patients in the class I, II, III, IV, or V groups were 20.0%, 5.6%, 13.3%, 17.4%, and 45.7%, respectively. 209 (95.9%) were discharged to a level of care defined as adequate or above-adequate by the PSI. Of the nine patients who were not discharged in this manner, one died within 28 days after ED admission (Table2B).

The estimated odds of death within 28 days increased 2.1-times with each point increase in PSI score (OR, 2.09; 95% CI, 1.32–3.32) (Table3). PSI class (I, II, or III vs. IV or V) predicted pneumonia-related mortality with an overall sensitivity and specificity of 82% and 34%, respectively (Table4). ROC analysis revealed that although the PSI's diagnostic performance was significantly better than chance (*P *< 0.001), the PSI had only modest utility for discriminating between fatal and nonfatal pneumonia cases (AUC, 0.6582; 95% CI, 0.57–0.75).

### Other independent factors predicting 28-day mortality

Univariate analysis revealed that, in addition to CURB-65 and PSI scores, RT in the 4 weeks before ED presentation was significantly associated with 28-day mortality. Renal disease was similarly associated with 28-day mortality, however, this variable is included in the PSI score. (Table3). The risk of death for patients receiving RT in the prior 4 weeks was four times higher than that of patients who had not (OR, 4.07; 95% CI, 1.47–11.28). Risk of death within 28 days was not significantly associated with age, gender, SIRS status, tumor type, leukemia, absolute neutrophil count, steroid use, the presence of liver disease, cerebrovascular accidents, altered mental status, antibiotic use, chemotherapy, immunotherapy, novel agents, SCT, or pneumonia classification.

**Figure 1:**
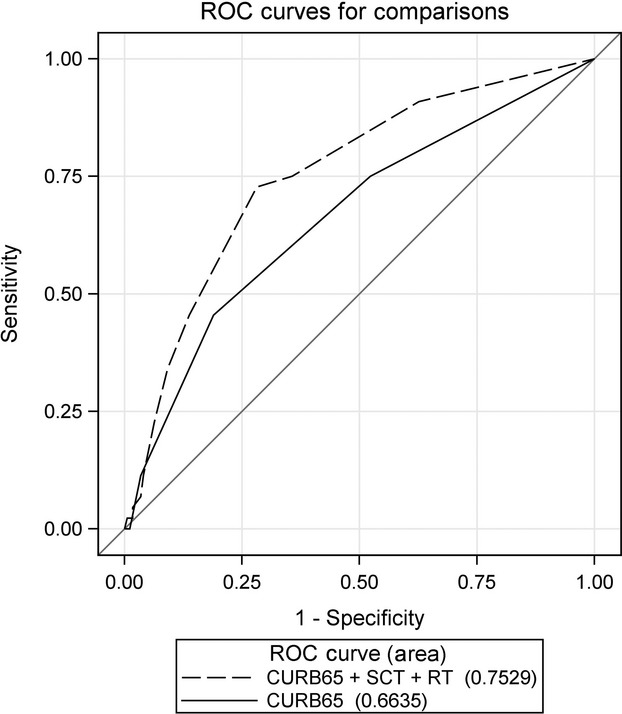
Receiver operating characteristics curves. Displayed are the areas under the curve (AUCs) for the model that included all three predictors (CURB-65 score, RT, and SCT) and CURB-65 score alone.

**Figure 2:**
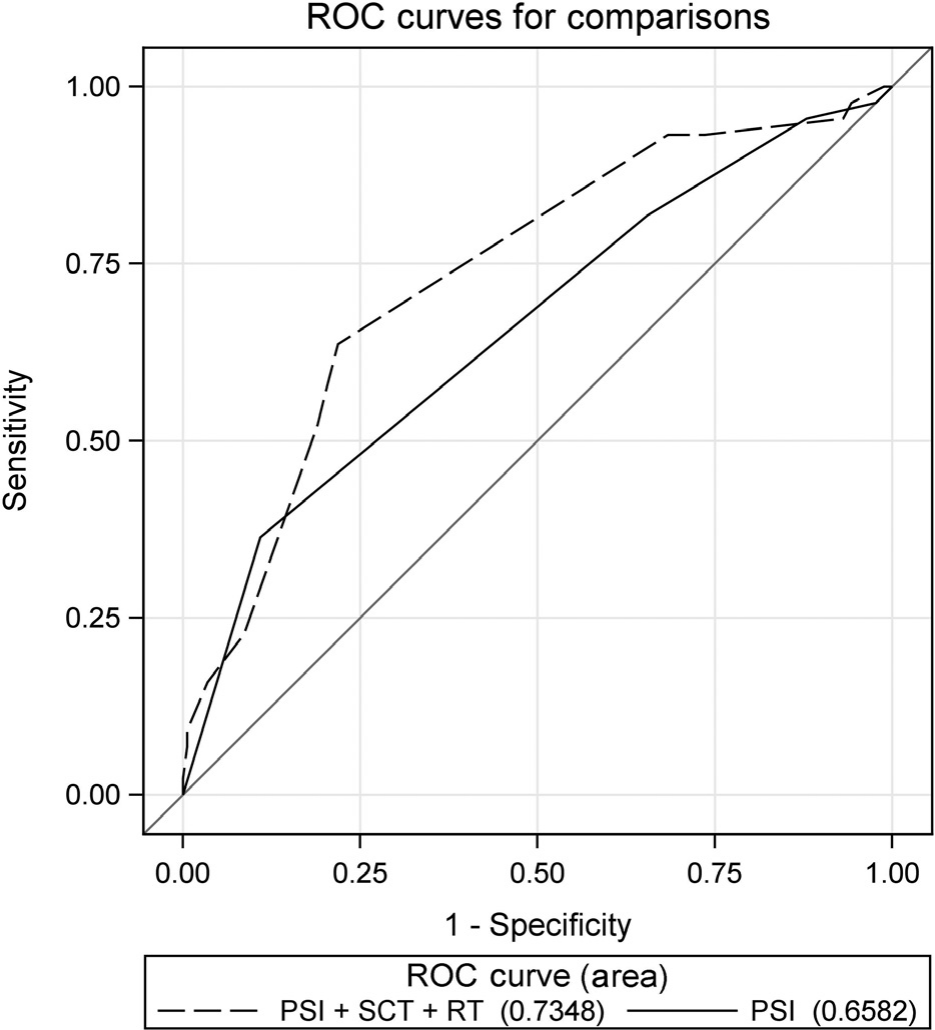
Receiver operating characteristics curves. Displayed are the areas under the curve (AUCs) of the model that included all three predictors (PSI score, RT, and SCT) and the PSI score alone.

In the multivariate logistic regression analysis that included the CURB-65 score, RT and SCT were significant predictors of 28-day mortality. Comparing the AUC for 28-day mortality prediction of the CURB-65 score alone with that of the model including CURB-65 score, RT, and SCT (AUC, 0.75; 95% CI, 0.67–0.83) revealed that the three-variable model predicted 28-day mortality significantly better than CURB-65 score alone did (*P *= 0.048) (Table5, Fig.1). Similarly, in the multivariate model including the PSI score, recent RT and SCT were significant predictors of 28-day mortality. The AUC for 28-day mortality prediction of the PSI score alone compared with that of a model including PSI score, RT, and SCT (AUC, 0.74; 95% CI, 0.65–0.82) revealed that the three-variable model predicted 28-day mortality significantly better than PSI score alone did (*P *= 0.043) (Table5, Fig.2).

**Table 5:** Receiver operating characteristic (ROC) association statistics and contrast test.

ROC model	AUC	SE	95% CI	AUC difference	*P*
CURB-65 + RT + SCT	0.7529	0.0402	0.6742–0.8317	0.0894	0.0480
CURB-65 alone	0.6635	0.0453	0.5748–0.7523		
PSI + RT + SCT	0.7348	0.0409	0.6547–0.8150	0.0766	0.0430
PSI alone	0.6582	0.0445	0.5709–0.7456		

AUC, area under the curve; SE, standard error; CI, confidence interval.

## Discussion

The first aim in this study was to determine whether the CURB-65 and PSI could be used to predict 28-day mortality in cancer patients diagnosed with pneumonia at a comprehensive cancer center's ED. We found that a CURB-65 score of ≥2 and a PSI score >III were significantly associated with an increased rate of 28-day mortality, and at a higher percentage per score compared with published data. However, ROC analysis revealed that both the CURB-65 and PSI discriminated poorly between fatal and nonfatal pneumonia. This finding is consistent with those of Aliberti et al. who studied 280 cancer patients with CAP 14, and with Jeong's study 15.

In the present study, the 28-day pneumonia-related mortality rate of patients categorized by CURB-65 scores <2 or low risk, were associated with a higher risk of death of 14.6% than has been reported in other settings (<2%) 6. Similarly, for those patients categorized by the CURB-65 as intermediate- or high risk (35.7% and 45.5%, respectively), the mortality rates were higher than those reported elsewhere (9.2% and 22.0%, respectively). In addition, 150 (68.8%) of the 218 patients in the present study received disposition that was not in line with that recommended in the CURB-65 guidelines. For instance, 144 patients categorized by the CURB-65 as low-risk were hospitalized, which is significantly higher than that recommended in the CURB-65 guidelines and suggests that the attending physician's clinical judgment often differed from the guidelines' recommendations.

Compared with our study population, the patient population with which Lim et al. developed the CURB-65 6 had scores that indicated a higher risk of pneumonia-related mortality. In that study, 98% of patients had a score of 4 or 5, whereas in the present study, less than 1% of patients had a score of 4, and none had a score of 5. Lim et al. 6 found that the sensitivity and specificity of a CURB-65 score of ≥2 in predicting pneumonia-related mortality were 75% and 69%, respectively. However, this cut-off score did not perform as well in the present study, in which a CURB-65 score of ≥2 predicted pneumonia-related mortality with an overall sensitivity and specificity of 45% and 81%, respectively. The discrepancies between our findings and those of the CURB-65 developers may be attributable to the different study populations, which excluded patients with solid organ and hematologic malignancy as well as those patients that were immunocompromised.

In contrast to the CURB-65, the PSI categorized 96% of the patients in the present study to receive an adequate level of care as recommended by the guidelines. Compared with the CURB-65, the PSI had a higher sensitivity in predicting mortality and classified a higher proportion of patients as high risk. Although the mortality rates of the patients in each of the risk classes in the present study are higher than those found by Fine et al. 7, in both studies, the mortality rate increased with increasing PSI risk class. The PSI has been most recently evaluated in other immunocompromised patients 16 and HCAP patients 17. Sanders et al. found that the mortality rate (20%) and PSI scores of patients who were immunocompromised because of hematologic malignancies, chemotherapy, RT, or SCT were higher than those of patients whose immunosuppression was due to HIV, solid organ transplant, or immunosuppressive drugs. Carraba et al. found that among immunosuppressed HCAP patients, the PSI had high sensitivity, but poor specificity in predicting mortality.

In our study, 87.6% of the patients had HCAP, and most were immunocompromised. The microbiology identified in the cultures obtained in our study was consistent with pathogens identified by Kollef et al. 18,19 in patients with HCAP. Similarly, there was an increased rate of drug-resistant pathogens in this group, which suggests that the patients in our study constituted a population that was much more complicated and ill than those with which the CURB-65 and PSI were developed.

Our second objective was to identify other variables that could predict an increase in 28-day mortality. Univariate analysis revealed that age, SIRS criteria, tumor type, neutropenia status,, steroid use, chemotherapy, immunotherapy, novel agents, and SCT status were not significantly associated with an increased risk of 28-day mortality. The presence of pulmonary infiltrates in patients with profound neutropenia has been associated with a higher risk of mortality 20. However, like Alberti et al. 21 and Joos et al. 22, we did not find an association between neutropenia or neutropenia severity and increased 28-day mortality. The ED's aggressive antimicrobial protocol for managing patients with febrile neutropenia and/or sepsis could account for this finding. We did find that the mortality risk of patients who received RT in the 4 weeks prior to ED presentation was significantly higher than that of patients who had not. Although Sanders et al. 16 also identified an increased mortality risk in cancer patients who received RT, whether that factor represented a statistically significant predictor of mortality, as our study did, remains unclear.

There are no tools to assess pneumonia severity in cancer patients specifically. We found that including two additional risk factors—recent RT and SCT—with the CURB-65 or PSI predicted 28-day mortality more effectively than either scale did alone. The CURB-65, when combined with RT and SCT, increased the AUC from 0.66 (95% CI, 0.57–0.75) to 0.75 (95% CI, 0.67–0.83), a significant improvement (*P *= 0.048). Likewise, the PSI, when combined with RT and SCT, produced a significant increase in the AUC, from 0.66 (95% CI, 0.57–0.75) to 0.73 (95% CI, 0.65–0.82) (*P *= 0.043).

Our study was not without potential limitations, one of which was the small sample size, which may have resulted in misleading mortality rates in the low-risk groups. Another limitation is that we did not include patients' disease stages in our analysis. However, only 12 patients were sent to hospice, and this could be an indicator of the patients being terminally ill. Nor did we include the number of patients admitted to the hospital floor for comfort measures only, which could be an important factor to consider when developing tools to evaluate pneumonia risk in cancer patients. Some potential pathogens may have been missed because other than cultures, some of the diagnostic procedures such as paired sera, urinary antigen test, rapid antigen test for influenza or polymerase chain reaction (PCR) used to identify the etiology of pneumonia were not routinely performed in our study. A further limitation was the retrospective nature of the study, as selection bias could have influenced the significance of our findings. Nevertheless, to our knowledge, this is one of the few studies to examine factors that predict mortality from pneumonia in cancer patients.

Clinicians should consider that the 28-day mortality rate of cancer patients with pneumonia is higher than that of noncancer patients with pneumonia. The results of our study suggest that the CURB-65, which classified the majority of patients as low-risk, is not a good predictor of 28-day mortality in cancer patients. The 28-day mortality rate of cancer patients who had received RT within 4 weeks of presenting to the ED with pneumonia had a higher risk of 28-day mortality, and physicians should consider this when making clinical decisions. The PSI was more sensitive than the CURB-65 in predicting 28-day mortality and may serve as a better tool for assessing the risk of pneumonia-related mortality in cancer patients. It is possible that the that lung injury induced by RT to the chest and prolonged immunosuppression in patients post SCT are the mechanisms underlying mortality and indirectly measured by the PSI. Neither the CURB-65 nor PSI should serve as a substitute for clinical judgment.

Further research to identify objective clinical factors that could discriminate between fatal and nonfatal pneumonia in cancer patients who present to the ED is warranted. Any model resulting from such research would have to be validated in an independent cohort of patients. Prospective studies to determine whether the risk factors of RT and SCT affect 28-day mortality in cancer patients are also important.

In conclusion, cancer patients, particularly SCT recipients and patients who received RT in the 4 weeks prior to presentation to an ED with pneumonia, are at a high risk of dying from pneumonia. The inadequate performances of the CURB-65 and PSI in our patient population is demonstrated in this study. We found that the addition of RT and SCT to the PSI improves the sensitivity of this instrument. Further studies are needed to develop a tool that assesses pneumonia-related mortality risk in cancer patients and immunocompromised patients.

## Conflict of Interest

None declared.
